# Social determinants of multimorbidity in older adults in sub-saharan Africa: A systematic review

**DOI:** 10.1017/S1478951525100576

**Published:** 2025-08-26

**Authors:** Sunkanmi Folorunsho, Raji Abdullateef, Medinah Suleiman, Munirat Sanmori

**Affiliations:** 1 Department of Sociology, University of Nebraska-Lincoln, Lincoln, NE, USA; 2 Department of Sociology, University of Ilorin, Ilorin, Nigeria; 3 Department of Common and Islamic Law, University of Ilorin, Ilorin, Nigeria; 4 Department of Sociology, Georgia State University, Atlanta, Georgia

**Keywords:** multimorbidity, social determinants, sub-saharan Africa, older adults, health disparities, chronic diseases

## Abstract

**Background:**

Multimorbidity is increasingly common among older adults in Sub-Saharan Africa (SSA), yet the role of social determinants in shaping its prevalence and outcomes remains underexplored.

**Objectives:**

This review aimed to (a) identify the prevalence, types, and patterns of multimorbidity among older adults in SSA; (b) examine the influence of social determinants such as income, education, healthcare access, and geographic location; (c) evaluate current approaches for prevention and management; and (d) propose directions for future research.

**Methods:**

A systematic search of six databases (PubMed, EMBASE, PsycINFO, Google Scholar, CINAHL, and Global Index Medicus) was conducted to identify quantitative studies published between 2000 and 2024 on adults aged 50 and above. Of 841 records screened, 16 studies met inclusion criteria and passed quality appraisal. The review protocol was registered in PROSPERO (CRD42024607875).

**Results:**

Multimorbidity ranged from 5.4% in Botswana to 71% in Nigeria. Cardiometabolic conditions often co-occurred with infectious and mental disorders. Poverty and low education significantly increased risk (OR: 1.44–7.44). Rural residents faced limited healthcare access, while urban dwellers had higher risks from lifestyle factors. Obesity and food insecurity further heightened vulnerability, especially among women and older adults.

**Significance of Results:**

Findings indicate that social determinants critically shape multimorbidity risk and outcomes in SSA. Integrated care models, targeted interventions, and policies addressing structural inequalities are urgently needed. Future research should apply longitudinal and qualitative approaches to clarify causal pathways and inform context-sensitive strategies.

## Introduction

A major issue within the African health literature is the lack of research examining how social determinants contribute to multiple health problems, especially among older adults (Azevedo 2017). While there is a substantial body of literature in advanced countries investigating how various social determinants combine to influence multimorbidity, such studies are notably lacking in Sub-Saharan African (SSA) (Alaba and Chola 2013; Arokiasamy et al 2015). SSA is the region of the African continent located south of the Sahara Desert, encompassing all African countries outside of North Africa. This region is currently experiencing significant population aging, as the global population reaches unprecedented levels. According to the United Nations, the number of older adults in SSA is expected to grow from around 46 million in 2020 to nearly 157 million by 2050, marking one of the fastest aging populations globally (United Nations 2020). Although this demographic trend is expected, it implies an increase in the occurrence of multiple health problems linked to social determinants.

Multimorbidity is commonly conceptualized as the presence of two or more chronic conditions in an individual (Skou et al 2022; Tong et al 2024). This phenomenon has far-reaching consequences for individuals, families, healthcare systems, and society, particularly in SSA, where limited resources make managing multiple chronic diseases especially challenging (Koné Pefoyo et al 2015). Unlike comorbidity, which examines how additional conditions impact a primary disease (Skou et al 2022), multimorbidity adopts a holistic, patient-centered approach aimed at addressing all of a patient’s conditions. However, in practice, treatment often prioritizes the most pressing or severe health issues, reflecting the constraints faced by both patients and healthcare providers (Tugwell and Knottnerus 2019). The implications of multimorbidity are far-reaching. Individuals dealing with multiple chronic conditions face a higher risk of premature death (Navickas et al 2016), are more frequently hospitalized (Rodrigues et al 2022) and tend to have longer hospital stays compared to those managing only one chronic illness (Skou et al 2022).

Social determinants, including income, education, living conditions, and access to healthcare, are key drivers of health disparities and chronic disease patterns among older adults in SSA (Arokiasamy et al 2015). Despite their critical role, there remains a considerable gap in understanding how inequalities related to these determinants shape the patterns of multimorbidity, particularly among older adults (Álvarez-Gálvez et al 2023). While it is well understood that lower socioeconomic status increases the risk of chronic diseases, literature falls short in adequately defining the relationships between the various chronic conditions that characterize multimorbidity and the social factors that drive them (Hien et al 2014). More specifically, to the best of our knowledge, no known research to date has systematically examined how these determinants of multimorbidity affect older adults across multiple countries in SSA. This is a critical gap, as socio-economic conditions, healthcare systems, and policies vary significantly between countries, potentially leading to differences in how multimorbidity manifests and progresses. The lack of cross-national studies leaves an incomplete understanding of how social determinants contribute to the development of multimorbidity. The urgency of addressing these gaps is clear. Multimorbidity disproportionately affects the disadvantaged population of older adults in this region, and without a comprehensive understanding of the social determinants at play, policy efforts aimed at reducing health inequalities will remain insufficient.

This systematic review seeks to address gaps in literature by pursuing the following objectives: (a) identifying the prevalence, types, and patterns of multimorbidity among older adults in SSA; (b) examining the social determinants that influence multimorbidity; (c) evaluating approaches for the prevention and management of multimorbidity; and (d) exploring future research directions in social determinants and multimorbidity research.

### Theoretical perspective

The relationship between social determinants and multimorbidity among older adults in SSA can be understood through the lens of General Strain Theory (GST). This theory provides a framework for examining how chronic socioeconomic stressors, such as poverty, housing instability, and food insecurity, create pathways that lead to adverse health outcomes (Agnew, 1992). Drawing from stress and coping paradigms, GST explains how prolonged exposure to strain disrupts biological systems and fosters maladaptive health behaviors, ultimately amplifying the risk of multimorbidity in vulnerable populations (Kilpatrick et al 2003). GST posits that the persistent socioeconomic disadvantages many older adults in SSA face represent a “first phase” of strain, akin to what Patterson (1982) describes as “basic training” for maladaptive coping. For example, early-life conditions of undernutrition, poor access to education, and inadequate healthcare set the stage for chronic vulnerability. These conditions are further aggravated by systemic inequities, such as limited healthcare infrastructure and social exclusion, which reinforce cycles of deprivation. These early stressors often lead to biological wear and tear, reflected in elevated risks for chronic conditions like hypertension, diabetes, and cardiovascular diseases (McEwen and Stellar 1993).

Once older adults encounter the compounding effects of poverty and limited access to healthcare, they enter what could be termed an “advanced phase” of strain, where their capacity to manage chronic conditions becomes increasingly compromised. This is particularly pronounced in rural SSA, where healthcare resources are sparse and healthcare-seeking behaviors are hindered by economic constraints (Eriksson et al 2014). The stress of managing multiple chronic conditions without adequate support or resources perpetuates a cycle of health decline, creating what Caspi et al (1987) describe as “cumulative continuity.” This concept aligns with the sustained and self-reinforcing nature of health disparities seen in multimorbidity.

Materialist perspectives further illuminate how inequitable access to essential resources drives disparities in multimorbidity outcomes (Braveman et al 2011; Marmot 2005). These theories argue that systemic barriers such as lack of access to nutritious food, clean water, and quality healthcare establish a context where chronic illnesses are not only prevalent but also poorly managed. For instance, in SSA, individuals from low-income households are more likely to experience malnutrition and delayed treatment for preventable diseases, compounding their health risks. These resource constraints are particularly severe for older adults, who often lack financial independence and rely on strained familial or community support systems.

While GST and materialist theories reveal the challenges older adults face, resilience frameworks provide a counterpoint by focusing on the protective factors that can mitigate these strains (Ungar 2011). Resilience in SSA is often rooted in strong social and familial networks, community solidarity, and culturally embedded coping mechanisms. These factors can buffer against the impacts of strain, promoting adaptive health behaviors even in resource-limited settings. For example, community health initiatives that emphasize regular health screenings, nutrition education, and physical activity have been shown to reduce the burden of multimorbidity in some SSA populations (Wister et al 2022).

## Method

### Literature and eligibility criteria

We adhered to PRISMA guidelines to ensure a structured and transparent methodology for this systematic review (Page et al 2021). Our primary search terms included “*multimorbidity,” “chronic conditions,” “social determinants,” “socioeconomic factors,” “older adults,” and “Sub-Saharan Africa*.” To capture region-specific trends, we incorporated additional keywords such as “*multimorbidity in Nigeria*,” “*chronic conditions in Kenya*,” and “*social determinants in Ghana*.” Boolean operators (e.g., “AND,” “OR”) were applied to refine the search strategy. We restricted the search to studies published in English from 2000 onward, aligning with our objective of examining social determinants and multimorbidity in aging populations. We focused on quantitative studies to align with our review’s objective of evaluating prevalence, risk ratios, and patterns using standardized measures. Studies were included if they:

- Addressed multimorbidity among adults aged 50 and above in SSA, defining multimorbidity as the coexistence of two or more chronic conditions (e.g., non-communicable diseases, mental health conditions, or communicable diseases like HIV or tuberculosis).
- Investigated associations between multimorbidity and social determinants (e.g., socioeconomic status, education, healthcare access) to explore their influence on health outcomes.
- Utilized cross-sectional, longitudinal, or case-control designs with established multimorbidity metrics.

We excluded studies published before 2000 to ensure relevance to current healthcare trends and demographic shifts. Qualitative studies were excluded due to our focus on quantitative analyses. Grey literature, such as conference proceedings or policy reports, was not systematically included due to potential limitations in methodological rigor. By focusing on quantitative evidence, we aimed to provide robust and measurable insights into multimorbidity patterns in SSA.

### Screening and selection process

We conducted a comprehensive search across multiple databases, including PubMed, Google Scholar, PsycINFO, ProQuest, Ageline, EBSCO, Embase, CINAHL, Global Index Medicus, and DARE. Our search yielded 841 studies. One independent reviewer screened titles and abstracts to ensure accuracy, resolving discrepancies through consensus or consultation with a second reviewer. This double-screening process reduced the pool to 152 relevant studies. We then conducted a full-text review, narrowing the selection to 65 studies that met our inclusion criteria. During this stage, 32 studies were excluded due to poor data quality or irrelevant outcomes. Ultimately, 16 studies were included in our final review, forming the basis for a comprehensive analysis.

### Data extraction and quality assessment

We employed a standardized protocol for data extraction. Key details we collected included:

As Identified in the Table 1, data synthesis involved examining prevalence rates and odds ratios, where possible, to identify patterns and differences across groups. This approach ensured a more nuanced understanding of how multimorbidity impacts health outcomes in varying socioeconomic and geographic settings. Statistical methods were tailored to aggregate prevalence estimates and compare key metrics across diverse study populations, enhancing the robustness of our findings.

**Table 1.** Standardized protocol

Study Characteristics	Author(s), year, geographic location (city/country), and study design
Population Data	Sample size, age range, follow-up duration, and institutionalization status
Multimorbidity Metrics	Definitions (e.g., ≥ 2 or ≥ 3 chronic conditions), prevalence rates, and associated healthcare metrics (e.g., hospitalizations, readmissions).
Outcome Metrics	Prevalence rates, odds ratios (ORs), and 95% confidence intervals (CIs).

### Ethical considerations

While no ethical approvals were required for the review itself, we adhered to PROSPERO guidelines to ensure transparency and accountability throughout the process. We acknowledged potential biases related to language and publication year restrictions and sought to mitigate these by employing comprehensive search terms and a rigorous double-screening process. We also discussed limitations, such as reliance on self-reported data and variability in multimorbidity definitions, in subsequent sections.

### Search strategy and study selection

**Figure 1.**
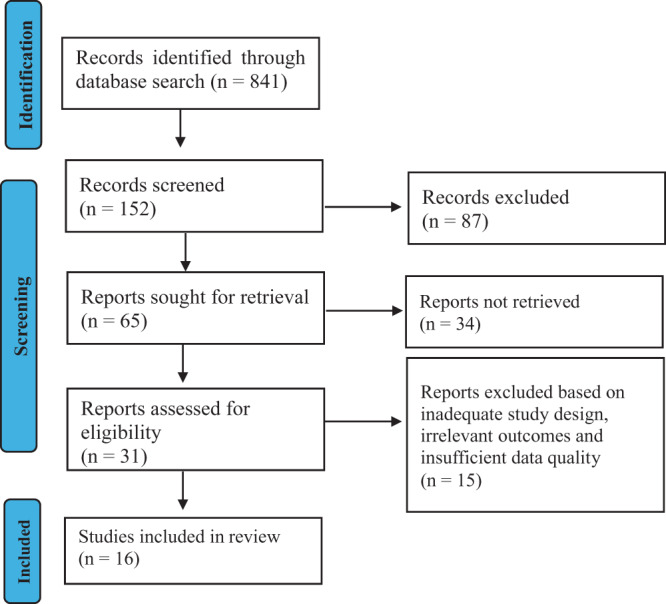
PRISMA flowchart of selected studies.

Our systematic search identified 841 initial records (see Figure 1). Following abstract screening, 152 studies remained relevant. A thorough full-text review further refined this to 65 studies that met all inclusion criteria. After excluding studies with methodological flaws or inadequate reporting, 16 studies were selected for final inclusion. These studies represented diverse geographic regions and included both urban and rural settings. This study was registered with PROSPERO (ID: CRD42024607875),

## Results

### Characteristics of the included studies

Sixteen studies were included in this review, spanning multiple African countries and covering a wide range of study designs, populations, and determinants (Table 2). Most studies (81%) used cross-sectional designs, while three employed longitudinal approaches (Eyowas et al 2023; Nwani and Isah 2016; Tomita et al 2021). The sample sizes ranged widely, from 240 participants in Sierra Leone (Lakoh et al 2023) to 3,889 participants in South Africa (Chang et al 2019). The studies predominantly focused on older adults, with minimum age thresholds of 50 years or older, consistent with the review’s emphasis on multimorbidity in aging populations. The study populations were diverse, including urban elderly populations (Hien et al 2014; Mohamed et al 2021), rural communities (Abdulraheem et al 2017; Musa et al 2024), and specific subgroups such as urban slum dwellers (Mohamed et al 2021) or hospital inpatients (Nwani and Isah 2016). Sampling methods varied, with random sampling employed in six studies (e.g., Chang et al 2019; Keetile et al 2020) and stratified sampling in others (e.g., Baamlong et al 2013; Eyowas et al 2022).

**Table 2.** Characteristics of articles examined for study

Author (Year)	Country	Design	Study Population	Sample Size and Sampling Methods	Outcome Variable	Data Source for Outcome	Determinants Examined	Data Source for Determinants
Hien et al (2014)	Burkina Faso	Cross-sectional	Urban elderly, age ≥ 60	712, Census sampling	Multimorbidity prevalence	Medical records, clinical exams	Age, Sex, Comorbidity patterns	Interviews, Medical history
Keetile et al (2020)	Botswana	Cross-sectional	Older adults aged 50 +	1,200, Random sampling	Multimorbidity	Primary data collection	Income, Health access	Structured surveys
Eyowas et al (2023)	Ethiopia	Longitudinal	Outpatients with chronic NCDs	1,123, Facility-based sampling	Multimorbidity, Hospitalizations, Mortality	Interviews, Medical record review	Age, QoL, Activation level	Standardized survey data
Eyowas et al (2022)	Ethiopia	Cross-sectional	Elderly, diverse urban areas	750, Stratified sampling	Chronic conditions	Health records	Socioeconomic status, Education	Questionnaires
Nimako et al (2013)	Ghana	Cross-sectional	Adults attending an urban clinic	1,527, Convenience sampling	Multimorbidity prevalence and disease pairs	Structured questionnaires	Age, Sex, Family history	Interviews, Health records
Ayanore et al (2024)	Ghana	Cross-sectional	Urban adults aged 60 +	1,100, Stratified sampling	Multimorbidity	Primary data collection	Income, Education, Healthcare access	Survey and demographic data
Mohamed et al (2021)	Kenya	Cross-sectional	Urban slum adults, age 50-60	2,003, Random sample within slums	Lifetime and episodic multimorbidity	Self-report, Biomarkers, Anthropometry	Age, Gender, Employment, Lifestyle factors	Survey, Demographic health surveillance
Nwani and Isah (2016)	Nigeria	Prospective	Elderly inpatients, age ≥ 65	345, Systematic sampling	Multimorbidity, Chronic disease patterns	WHO definition, Medical record review	Age, Sex, Disease type, Healthcare use	Longitudinal patient data
Abdulraheem et al (2017)	Nigeria	Cross-sectional	Rural elderly, age ≥ 60	1,650, Cluster sampling in rural areas	Multimorbidity prevalence, Chronic conditions	Questionnaire, History taking, Medical records	Age, Socioeconomic status, Rural setting	Structured interviews and questionnaires
Baamlong et al (2013)	Nigeria	Cross-sectional	Older adults in urban clinics	850, Stratified sampling	Multimorbidity	Clinical exams	Health access, Income	Surveys and medical records
Lakoh et al (2023)	Sierra Leone	Cross-sectional	Adults with TB in a tertiary hospital	240, Non-random sampling	TB multimorbidity	Clinical assessments at Connaught Hospital	Age, Gender, Comorbid diseases	Questionnaire, Clinical measurements
Chang et al (2019)	South Africa	Cross-sectional	Rural adults aged 50 +	3,889, Random sampling	Multimorbidity	HAALSI Program survey	Socio-demographics, Wealth, HIV status	Health and Ageing in Africa: HAALSI
Waterhouse et al (2017)	South Africa	Cross-sectional	Older adults aged 50 +	3,842, Nationally representative sample	Disability due to multimorbidity	South African Study on Global Ageing and Health	Chronic diseases, Hypertension, Wealth	South African Study on Global Ageing and Health (SAGE)
Musa et al (2024)	Sudan	Cross-sectional	Adults in three villages, Wad Hamid district	250, Systematic sampling	Prevalence of multimorbidity	Community survey	Age, Gender, Obesity, Socio-demographics	Household interviews
Tomita et al (2021)	Tanzania	Cross-sectional	Urban adults aged 50 + in Dar es Salaam	2,299, Population-based cohort	Multimorbidity and Hospitalization	Dar es Salaam Health and Demographic Surveillance	Socio-economics, Food insecurity	Health Demographic Surveillance System (HDSS)
Bulamba et al (2024)	Uganda	Cross-sectional	Adults aged 55 + in Wakiso	604, Population-based survey	Mental health and C-NCDs	AMBSO Population Health Surveillance	Socio-demographics, Depression, Anxiety	Questionnaire using PHQ-9 and GAD-7

### Types of multimorbidity

Multimorbidity patterns reported in the studies indicated the prevalence of chronic cardiometabolic conditions, mental health disorders, and infectious diseases (Table 3). Cardiovascular conditions such as hypertension were most frequently reported across all regions, often in combination with diabetes or other metabolic disorders (Hien et al 2014; Nimako et al 2013). Mental health disorders were prevalent in several studies, especially when co-occurring with chronic non-communicable diseases (Bulamba et al 2024; Tomita et al 2021). Specific contexts, such as urban slums, reported high multimorbidity involving respiratory and infectious diseases, including tuberculosis (Lakoh et al 2023; Mohamed et al 2021). Some studies identified unique clusters, such as “cardio-mental” or “metabolic-respiratory” patterns, reflecting localized health dynamics and data stratification (Chang et al 2019; Eyowas et al 2022). The interplay of mental health and chronic diseases was particularly pronounced in Uganda and Tanzania, where depression and anxiety were frequently linked with multimorbidity (Bulamba et al 2024; Tomita et al 2021).

**Table 3.** Multimorbidity types based on selected articles

Author (year)	Country	Types of Multimorbidity
Keetile et al (2020)	Botswana	Cardiovascular, metabolic, and respiratory diseases
Hien et al (2014)	Burkina Faso	Hypertension, malnutrition, visual impairments, diabetes, osteoarthritis, dementia
Eyowas et al (2023)	Ethiopia	Cardiovascular, metabolic, mental, and respiratory conditions
Eyowas et al (2022)	Ethiopia	Four main patterns: cardiovascular, cardio-mental, metabolic, respiratory clusters
Nimako et al (2013)	Ghana	Hypertension with diabetes, musculoskeletal issues, cardiovascular conditions
Ayanore et al (2024)	Ghana	COVID-19 study on healthcare utilization and chronic condition interactions
Mohamed et al (2021)	Kenya	High multimorbidity in urban slums; includes chronic respiratory, metabolic, and infectious diseases
Nwani and Isah (2016)	Nigeria	Circulatory, endocrine, and metabolic diseases most common; defined as two or more chronic diseases
Abdulraheem et al (2017)	Nigeria	Commonly includes hypertension, diabetes, osteoarthritis, visual impairments, and malnutrition
Baamlong et al. (2013)	Nigeria	High prevalence in cardiovascular and musculoskeletal diseases, often involving metabolic disorders
Lakoh et al (2023)	Sierra Leone	Tuberculosis multimorbidity with common comorbidities like hypertension, diabetes, and HIV
Chang et al (2019)	South Africa	Combination of cardiometabolic conditions, HIV, anaemia, and mental health issues among older adults
Waterhouse et al (2017)	South Africa	Hypertension, musculoskeletal issues, respiratory conditions, often linked to disability
Musa et al (2024)	Sudan	High prevalence of diabetes, hypertension, anaemia, obesity, and mental health disorders (depression/anxiety) among adults
Tomita et al (2021)	Tanzania	Hypertension, HIV, diabetes, depression, stroke
Bulamba et al (2024)	Uganda	Chronic non-communicable diseases (e.g., diabetes, hypertension) co-occurring with mental health disorders (depression and anxiety)

### Social determinants of health

The studies examined in this review pointed to the critical role of social determinants of health (SDOH) in influencing multimorbidity patterns among older adults across diverse African contexts. Key determinants included socioeconomic status, education, healthcare access, gender, urbanization, and behavioral factors, all of which varied significantly across regions and demographic groups (Table 4).

**Table 4.** Social determinant types and measurement

Author (Year)	Country	Social Determinants	Measurement Method
Keetile et al (2020)	Botswana	Wealth, Education, Employment, Residence	Wealth as asset ranking index; education categorized by highest level attained (primary, secondary, tertiary); employment as binary (employed/unemployed).
Hien et al (2014)	Burkina Faso	Healthcare Access, Gender, Nutrition	Access categorized by rural/urban, gender binary, nutrition levels based on BMI categories.
Eyowas et al (2023)	Ethiopia	Socioeconomic Status, Physical Inactivity	Socioeconomic status ranked by income quintiles; Physical Activity Scale for Elderly (PASE).
Eyowas et al (2022)	Ethiopia	Age, Obesity	Age as continuous; obesity categorized by BMI (normal, overweight, obese).
Nimako et al (2013)	Ghana	Age, Education, Family History	Age as age bands (18–39, 40–49, 50–59, 60 +); education by years; family history as binary (yes/no).
Ayanore et al (2024)	Ghana	Financial Loss, Food Insecurity, Housing Instability	Financial loss binary (yes/no); food insecurity measured by Household Food Insecurity Access Scale; housing binary (stable/unstable).
Mohamed et al (2021)	Kenya	Employment, Alcohol Consumption	Employment as binary; alcohol use coded as current/past/non-drinker.
Nwani and Isah (2016)	Nigeria	Age, Socioeconomic Status	Age categorized (early elderly, late elderly); socioeconomic status ranked by household income quartiles.
Abdulraheem et al (2017)	Nigeria	Education, Gender, Urbanization	Education level (no education, primary, secondary, tertiary); gender binary; urbanization as rural/urban residence.
Baamlong et al. (2013)	Nigeria	Health Access, Gender, Family Support	Health access as utilization rate (low/high); gender binary; family support measured on a Likert scale.
Lakoh et al (2023)	Sierra Leone	Age, Gender, Hypertension, Diabetes, Obesity	Age as continuous; gender as binary (male/female); hypertension and diabetes diagnosed based on clinical assessment; obesity categorized by BMI.
Chang et al (2019)	South Africa	Wealth, HIV Status, Cardiometabolic Conditions	Wealth as tertiles; HIV status binary (positive/negative); cardiometabolic conditions assessed via clinical diagnostic criteria.
Waterhouse et al (2017)	South Africa	Disability, Hypertension, Socioeconomic Factors	Disability measured by WHODAS 2.0; hypertension binary (diagnosed/undiagnosed); socioeconomic factors as income categories.
Musa et al (2024)	Sudan	Age, Gender, Education, Employment	Age continuous; gender binary; education categorized (<secondary, ≥ secondary); employment binary (employed/unemployed).
Tomita et al (2021)	Tanzania	Food Insecurity, Hospitalization	Food insecurity binary (secure/insecure); hospitalization measured by hospital admission in past 12 months.
Bulamba et al (2024)	Uganda	Depression, Anxiety, Chronic Diseases	Depression measured by PHQ-9; anxiety by GAD-7; chronic diseases as self-reported diagnosis (e.g., hypertension, diabetes).

#### Socioeconomic status and wealth

Socioeconomic disparities were a prominent theme across multiple studies. Wealth indices and income quintiles were commonly used to assess the economic conditions of participants (Chang et al 2019; Keetile et al 2020). For instance, in Ethiopia, low socioeconomic status significantly increased the risk of multimorbidity, with an odds ratio (OR) of 1.90 (95% CI 1.30–3.00) (Eyowas et al 2023). Similarly, in South Africa, individuals in the lowest wealth tertile had a higher risk of multimorbidity (OR = 1.5, 95% CI 1.1–2.0) (Chang et al 2019). Financial distress related to COVID-19 in Ghana was also associated with a substantial increase in multimorbidity risk (AOR = 7.44, 95% CI 3.05–18.16) (Ayanore et al 2024).

#### Education

Education levels were consistently linked to multimorbidity prevalence. Lower levels of education were associated with increased risks, as evidenced by findings from Nigeria and Ghana. In rural Nigeria, participants with limited education faced higher odds of multimorbidity, amplifying their health vulnerabilities (Abdulraheem et al 2017). Similarly, in Ghana, years of education were inversely related to the prevalence of multimorbidity, emphasizing the protective role of educational attainment (Nimako et al 2013).

#### Healthcare access

Access to healthcare was a recurring determinant in several studies. Rural residents often experienced poorer health outcomes due to limited healthcare infrastructure and resources. For instance, rural elderly populations in Nigeria reported higher multimorbidity prevalence (68.4%) compared to their urban counterparts, highlighting the disparity in healthcare services (Abdulraheem et al 2017). In Sierra Leone, inadequate access to healthcare exacerbated tuberculosis-related multimorbidity, with comorbidities such as hypertension and diabetes being particularly prevalent (Lakoh et al 2023).

#### Urbanization and residence

The studies also emphasized the differential impacts of urbanization on health outcomes. Urban slum dwellers, such as those in Kenya, faced unique challenges including overcrowding, poor sanitation, and exposure to environmental hazards. These factors contributed to high multimorbidity rates (28.7%) among adults aged 40–60 years in urban slums, with unemployment (OR = 1.87, 95% CI 1.11–3.16) and current alcohol use (OR = 2.5, 95% CI 1.5–4.2) as significant contributors (Mohamed et al 2021). Conversely, rural populations faced higher risks due to limited healthcare access, as observed in South Africa and Nigeria (Abdulraheem et al 2017; Waterhouse et al 2017).

#### Behavioral and lifestyle factors

Behavioral determinants such as physical inactivity, obesity, and alcohol use emerged as critical contributors to multimorbidity. In Botswana, physical inactivity and obesity were significantly associated with multimorbidity, with obesity showing an adjusted odds ratio (AOR) of 1.44 (95% CI 1.12–2.61) (Keetile et al 2020). In Sudan, obesity and poor dietary practices were key risk factors for multimorbidity among rural populations, particularly among women (Musa et al 2024). Alcohol use was another notable determinant, especially in Kenya, where it was linked to elevated multimorbidity risks (Mohamed et al 2021).

### Prevalence of multimorbidity

The prevalence of multimorbidity varied widely across the studies, reflecting differences in population characteristics, healthcare access, and environmental factors (Table 5). The rates ranged from 5.4% in Botswana (Keetile et al 2020) to 71% in Nigeria (Baamlong et al 2013), showing the heterogeneity of health outcomes in African settings.

**Table 5.** Prevalence and odds of multimorbidity based on the selected articles

Author (Year)	Country	Prevalence of Multimorbidity	Odds Ratios and Results
Keetile et al (2020)	Botswana	5.4% prevalence among adults, with higher rates in women, overweight individuals, and alcohol consumers.	Alcohol use (AOR = 4.80, 95% CI 1.16–19.8); obesity (AOR = 1.44, 95% CI 1.12–2.61) showed strong association with multimorbidity (Botswana).
Hien et al (2014)	Burkina Faso	65% prevalence among the elderly, with age as a key factor.	Age ≥ 70 (AOR = 1.65, CI 1.01–2.68); rural residence and malnutrition were significantly associated with higher multimorbidity.
Eyowas et al (2023)	Ethiopia	A longitudinal study showed an increase in multimorbidity from 54.8% to 56.8% over one year among adults in outpatient clinics in Bahir Dar.	Low socioeconomic status (OR = 1.90, CI 1.30–3.00); physical inactivity linked to elevated multimorbidity risk.
Eyowas et al (2022)	Ethiopia	54.8% multimorbidity among adults attending outpatient care for chronic conditions in Bahir Dar.	Obesity (OR = 1.7, 95% CI 1.12–2.5); elderly individuals had significantly higher multimorbidity rates.
Nimako et al (2013)	Ghana	38.8% prevalence in urban clinics, mainly affecting those aged 40 + .	Age 50–59 (OR = 12.83, 95% CI 8.47–19.44) and family history (OR = 1.43, CI 1.16–1.78) were major contributors.
Ayanore et al (2024)	Ghana	Not specified	Financial distress due to COVID-19 (AOR = 7.44, 95% CI 3.05–18.16) significantly increased multimorbidity risk.
Mohamed et al (2021)	Kenya	28.7% multimorbidity in urban slums, with higher rates among older adults and the unemployed.	Unemployment (OR = 1.87, CI 1.11–3.16); current alcohol use (OR = 2.5, CI 1.5–4.2) linked with higher multimorbidity.
Nwani and Isah (2016)	Nigeria	49% multimorbidity in hospital inpatients, primarily those aged 65 + .	Low socioeconomic status (OR = 2.1, 95% CI 1.5–2.9); chronic conditions increased with age and income disparities.
Abdulraheem et al (2017)	Nigeria	68.4% among the rural elderly, common in those aged 70 + .	Rural residents had a higher risk (OR = 1.72, CI 1.03–2.77) compared to urban areas; low education amplified multimorbidity risks.
Baamlong et al. (2013)	Nigeria	71% prevalence among general outpatients.	Limited healthcare access increased multimorbidity (AOR = 2.15, 95% CI 1.34–3.46); family support moderated health outcomes.
Lakoh et al (2023)	Sierra Leone	High rates of TB multimorbidity in a tertiary hospital; 47.9% with hypertension and 24.2% with diabetes among TB patients.	Older males had a higher risk of multimorbidity (OR = 1.9, 95% CI 1.4–2.8); diabetes and obesity elevated risks further.
Chang et al (2019)	South Africa	69.4% had two or more conditions; high HIV prevalence among rural older adults.	Low wealth (OR = 1.5, CI 1.1–2.0); HIV-positive individuals faced elevated multimorbidity rates.
Waterhouse et al (2017)	South Africa	13.2% among older adults with two or more chronic conditions.	Hypertension (OR = 2.2, 95% CI 1.5–3.2); disability rates higher among low-income groups with multiple chronic conditions.
Musa et al (2024)	Sudan	61.6% multimorbidity, highest among older and female participants.	Females (OR = 2.17, CI 1.16–4.06); low education increased multimorbidity, particularly among unemployed adults.
Tomita et al (2021)	Tanzania	25.3% prevalence in the urban population aged 40 +; higher hospitalizations linked to multimorbidity.	Food insecurity (OR = 1.8, 95% CI 1.2–2.5); frequent hospitalizations linked with multimorbidity.
Bulamba et al (2024)	Uganda	18.9% among older adults.	Depression (AOR = 2.10, CI 1.35–3.26); anxiety increased multimorbidity, particularly with chronic conditions.

#### Regional variations

In West Africa, studies reported some of the highest multimorbidity prevalence rates. For instance, in rural Nigeria, 68.4% of elderly participants reported multimorbidity, driven by limited healthcare access and educational disparities (Abdulraheem et al 2017). Similarly, in Ghana, multimorbidity prevalence was reported at 38.8% in urban clinics, with hypertension and diabetes as the most common conditions (Nimako et al 2013). In Sierra Leone, tuberculosis-related multimorbidity was prevalent, with 47.9% of TB patients also diagnosed with hypertension (Lakoh et al 2023). In East Africa, multimorbidity prevalence rates were also significant. In Ethiopia, 54.8% of adults attending outpatient care for chronic conditions reported multimorbidity, with obesity and low socioeconomic status as key contributors (Eyowas et al 2022). Kenya reported a 28.7% prevalence among urban slum populations, emphasizing the compounded risks of poverty and environmental hazards (Mohamed et al 2021).

In Southern Africa, the prevalence was relatively high in specific contexts. For example, 69.4% of participants in rural South Africa reported multimorbidity, with a notable burden of HIV-related conditions (Chang et al 2019). In urban Uganda, multimorbidity prevalence was 18.9%, with depression and anxiety significantly elevating risks (Bulamba et al 2024).

#### Demographic and behavioral influences

Age was consistently associated with higher multimorbidity prevalence. For instance, in Burkina Faso, participants aged ≥ 70 had significantly higher odds of multimorbidity (AOR = 1.65, 95% CI 1.01–2.68) (Hien et al 2014). Gender also played a role; women in Sudan were more likely to experience multimorbidity compared to men (OR = 2.17, 95% CI 1.16–4.06) (Musa et al 2024). Behavioral factors such as alcohol consumption and physical inactivity were particularly influential in Botswana and Kenya, where they significantly contributed to multimorbidity risks (Keetile et al 2020; Mohamed et al 2021).

## Discussion

Consistent with previous findings, the social determinants of health (SDOH) play a significant role in influencing multimorbidity among older adults in SSA. Studies reviewed in this analysis identified critical factors such as socioeconomic status, education, healthcare access, urbanization, and behavioral influences, which are consistent with broader global health research. For example, the strong association between low socioeconomic status and multimorbidity observed in Ethiopia (Eyowas et al 2023) aligns with findings from South Africa, where individuals in the lowest wealth tertiles experienced significantly higher multimorbidity rates (Chang et al 2019). These findings emphasize the profound impact of poverty on health outcomes, as limited financial resources restrict access to preventive care and health management options, perpetuating cycles of poor health.

Education levels also emerged as a consistent predictor of multimorbidity, particularly in settings where health literacy is essential for navigating healthcare systems. The association between lower education and increased multimorbidity risk, as seen in rural Nigeria (Abdulraheem et al 2017), mirrors findings from Ghana, where participants with fewer years of schooling faced heightened risks of developing multiple chronic conditions (Nimako et al 2013). These patterns highlight the protective role of education in promoting health literacy and empowering individuals to engage in health-promoting behaviors. Furthermore, healthcare access disparities further exacerbate multimorbidity risks, particularly in rural areas with limited infrastructure. For instance, rural elderly populations in Nigeria reported a multimorbidity prevalence of 68.4%, compared to lower rates in urban settings (Abdulraheem et al 2017). Conversely, urban slum residents in Kenya faced unique health risks due to overcrowding, poor sanitation, and environmental hazards, contributing to a multimorbidity prevalence of 28.7% (Mohamed et al 2021). These findings illustrate the complex interplay between geographic location and health outcomes, with both urban and rural environments presenting distinct challenges.

Behavioral factors such as obesity, physical inactivity, and alcohol use also showed a strong association with multimorbidity across multiple studies. In Botswana, obesity increased the risk of multimorbidity by 44% (Keetile et al 2020), while physical inactivity and unhealthy dietary habits were significant contributors in several settings. Alcohol use was notably impactful in Kenya, where it doubled the likelihood of multimorbidity (Mohamed et al 2021). These findings are consistent with global literature indicating the role of lifestyle factors in chronic disease clustering. Age and gender disparities in multimorbidity were also evident. Older adults consistently exhibited higher multimorbidity prevalence, as seen in Burkina Faso, where individuals aged 70 and above had significantly higher odds of developing multiple chronic conditions (Hien et al 2014). Gender differences were pronounced, with women in Sudan showing a greater likelihood of multimorbidity compared to men (Musa et al 2024). These findings suggest that biological, social, and cultural factors collectively contribute to these disparities.

Regional variations in multimorbidity prevalence across SSA further illustrate the heterogeneity of health outcomes. West Africa reported some of the highest prevalence rates, such as the 71% reported in urban clinics in Nigeria (Baamlong et al 2013). Similarly, in Sierra Leone, tuberculosis-related multimorbidity was prevalent, with 47.9% of TB patients also diagnosed with hypertension (Lakoh et al 2023). In East Africa, Ethiopia recorded a prevalence of 54.8%, driven by obesity and socioeconomic disadvantage (Eyowas et al 2022), while Southern Africa pointed to the intersection of HIV and other chronic conditions, as seen in rural South Africa (Chang et al 2019).

### Prevention and management of multimorbidity

The prevention and management of multimorbidity in SSA is an urgent public health challenge. With the increasing prevalence of chronic diseases and resource limitations in healthcare systems, preventive strategies are vital. Multimorbidity adversely affects morbidity, quality of life, and health system efficiency. Social determinants such as poverty, inadequate education, and geographic barriers exacerbate risks, indicating the need for culturally sensitive and accessible prevention interventions (Alaba and Chola 2013; Eyowas et al 2022)

#### Evidence-based prevention approaches

Prevention strategies targeting social determinants, lifestyle behaviors, and healthcare access are effective in reducing the onset of chronic conditions and multimorbidity. For example, community-based lifestyle interventions emphasizing physical activity, improved nutrition, and smoking cessation have reduced the incidence of cardiovascular and metabolic conditions in rural SSA populations (Eyowas et al 2022). In Ethiopia, a program promoting healthy diets and moderate physical activity among older adults in rural communities led to a 15% reduction in the prevalence of hypertension over two years (Eyowas et al 2023). These interventions are most impactful when combined with culturally tailored education on disease prevention (Chang et al 2019). For instance, localized health workshops in South Africa addressing the importance of balanced diets successfully increased vegetable intake and reduced sodium consumption among participants (Waterhouse et al 2017)

#### Task-sharing models

Task-sharing models involving community health workers (CHWs) have been considered as a promising solution to address prevention gaps caused by healthcare worker shortages. (Kok et al 2017). CHWs provide cost-effective, community-specific education and basic health services, particularly in underserved rural areas (Mossadeghi et al 2023). In South Africa, CHWs successfully implemented hypertension and diabetes education programs, which led to a 10% decrease in new diagnoses over a three-year period (Waterhouse et al 2017), this can be extended to other countries in SA. Similarly, CHWs in Tanzania improved compliance with lifestyle modifications among patients at high risk of diabetes by using culturally relevant narratives and community support systems (Tomita et al 2021). Scaling such programs could significantly reduce multimorbidity by providing preventive care for at-risk populations.

#### Integrated preventive strategies

Integrated strategies targeting the dual burden of infectious and non-communicable diseases are essential in SSA, where these diseases often co-occur. Programs addressing HIV, tuberculosis (TB), and diabetes simultaneously have shown significant success in reducing multimorbidity prevalence (Lakoh et al 2023). In Sierra Leone, an integrated approach involving health education and free screenings for TB, diabetes, and hypertension reduced the prevalence of untreated multimorbidity by 20% within two years (Lakoh et al 2023). Moreover, community-based education on adherence to HIV treatments has mitigated co-occurring conditions like metabolic syndrome in Uganda (Bulamba et al 2024).

#### Addressing socioeconomic and behavioral risks

Socioeconomic instability, food insecurity, and alcohol consumption are critical risk factors for multimorbidity in SSA. Interventions addressing these determinants are essential for prevention. Conditional cash transfer programs, such as those piloted in Kenya, improved access to nutritious food and preventive healthcare services, reducing diabetes-related complications by 25% (Mohamed et al 2021). Behavioral interventions like cognitive-behavioral therapy (CBT) for stress management have also shown potential in mitigating risk behaviors. For instance, CBT programs focusing on alcohol moderation in Botswana decreased risky drinking behaviors by 18%, thereby reducing associated hypertension cases (Keetile et al 2020).

#### Workforce and policy innovations

Policy-level initiatives to expand universal health coverage and enhance healthcare infrastructure are critical for multimorbidity prevention. Investments in primary healthcare systems, coupled with workforce development programs, improve access to preventive services. Mobile health (mHealth) platforms have shown promise in reaching remote populations (Cometto et al 2018). In Ghana, mHealth platforms providing teleconsultations and health education increased early detection of hypertension by 30% in rural regions (Ayanore et al 2024). Policy measures addressing social determinants, such as improving education access and creating employment opportunities, further reduce the risk of multimorbidity. For instance, vocational training programs in Nigeria increased household incomes, indirectly improving access to preventive healthcare (Abdulraheem et al 2017).

### Future research directions in social determinants and multimorbidity

#### Bridging data gaps

A comprehensive understanding of multimorbidity in SSA demands robust, region-specific datasets that capture the unique socio-economic, cultural, and health-related factors of the region. For instance, a study in Sierra Leone revealed how cultural perceptions of illness significantly influenced healthcare-seeking behavior among patients with conditions like tuberculosis and diabetes (Lakoh et al 2023). To build on such insights, future research should adopt mixed methods approaches that combine longitudinal data with ethnographic investigations. For example, integrating household health data with information on community resources, such as access to clean water or transportation, could provide useful predictors of disease progression. Moreover, datasets that link health outcomes with agricultural cycles could pinpoint periods of increased vulnerability, such as during food shortages or post-harvest seasons, enabling timely public health interventions. Moreover, longitudinal data would be particularly valuable in SSA, where the dynamic nature of multimorbidity is influenced by factors such as migration, conflict, and climate change. By tracking individuals over time, researchers can assess how shifts in income, education, or access to healthcare influence the onset and progression of multiple chronic conditions. For example, data from refugee camps in Uganda could illuminate how displacement affects long-term health outcomes, offering a framework for addressing multimorbidity in other crisis-affected regions.

#### Intersectionality approach

Multimorbidity in SSA is influenced by the intersection of gender, age, socioeconomic status, and geographic location. For example, research in Kenya shows that rural women with lower education levels face disproportionately higher rates of multimorbidity than their urban male counterparts, often due to limited healthcare access and traditional caregiving roles (Chang et al 2019). Future studies should probe into how these intersecting identities shape disease patterns and healthcare access. For instance, research could explore how patriarchal norms in rural settings influence women’s ability to seek timely medical care or how stigma associated with aging in some communities affects health outcomes for older adults. Analyzing intersectionality also allows for the design of targeted interventions. For example, interventions for women in rural Ethiopia could focus on integrating health education into agricultural cooperatives, where women often gather for financial and social support. Similarly, for young men in urban slums, interventions could prioritize addressing mental health and substance abuse issues linked to unemployment and social isolation. Understanding these complex interactions can guide the development of equitable healthcare policies and programs tailored to diverse population needs.

#### Policy implications and cross-sectoral approaches

Macro-level policies addressing education, economic stability, and healthcare infrastructure are foundational to reducing multimorbidity in SSA. For instance, policies that promote female literacy in Ethiopia have been linked to improved health outcomes, as educated women are more likely to access preventive services and manage chronic conditions effectively (Eyowas et al 2022). Research should evaluate how education reforms that increase access for marginalized groups impact long-term health outcomes. Furthermore, cross-sectoral collaborations could also play a pivotal role. For example, integrating health screenings into school feeding programs in Ghana addresses both educational and nutritional disparities simultaneously (Fernandes et al 2017). Similarly, policies promoting small-scale agricultural development could enhance food security while providing employment opportunities, indirectly reducing stress-related health behaviors. Future studies should explore these ripple effects to optimize policy frameworks.

#### Investigating technological innovations

Technological innovations offer transformative potential for managing multimorbidity in SSA. For instance, telemedicine platforms have been successfully deployed in Kenya, improving access to specialists for rural patients with chronic conditions (Chang et al 2019). Future research could evaluate the scalability of such platforms across other SSA regions, particularly when integrated with wearable health devices for real-time monitoring. Electronic health records (EHRs) could further improve the management of multimorbidity by enhancing care coordination across facilities. For example, a pilot EHR system in Rwanda allowed healthcare providers to track patients with diabetes and hypertension, reducing duplication of tests and improving treatment adherence. Future studies should explore how such systems can be adapted for use in low-resource settings, where infrastructure challenges remain significant.

### Conclusion and limitations

This systematic review accentuates the significant role social determinants play in shaping multimorbidity among older adults in SSA. Factors such as socioeconomic disparities, geographic location, and limited lifestyle options emerged as major influences on the prevalence and management of multimorbidity. Poverty, low levels of education, and restricted access to healthcare were shown to substantially increase the risk of chronic conditions and their clustering. Behavioral factors like alcohol consumption and physical inactivity further amplified these risks, often reflecting the harsh socioeconomic conditions in which many individuals in SSA live. The dual burden of non-communicable and infectious diseases in the region underscores the urgent need for healthcare models that address these unique and overlapping challenges.

That said, this review has its limitations. One key issue is the inconsistency in how multimorbidity was defined and measured across studies. While some studies considered only two coexisting conditions, others included a broader range of chronic diseases, making it challenging to compare findings. Most of the studies relied on cross-sectional data, which limits the ability to establish cause-and-effect relationships between social determinants and multimorbidity. Although patterns and associations were identified, the timing and progression of these factors remain unclear. Another limitation was the uneven geographic representation of the studies. Some regions of SSA were underrepresented, meaning the findings might not reflect the full diversity of experiences across the continent. Additionally, excluding qualitative research meant losing valuable insights into how individuals and communities perceive and cope with multimorbidity, particularly in resource-limited settings. Many studies also relied on self-reported data, introducing potential biases like underreporting, recall errors, or social desirability effects. Finally, focusing solely on peer-reviewed articles may have excluded relevant grey literature or studies with null findings, potentially narrowing the scope of this review.

Despite these limitations, this review makes meaningful contributions to understanding multimorbidity in SSA. It shows how poverty, limited education, and unequal access to healthcare are deeply intertwined with health outcomes in older adults. It also draws attention to the pressing need for integrated healthcare solutions to address the dual burden of non-communicable and infectious diseases in the region. Future research should aim for consistent definitions and measurements of multimorbidity to improve comparisons across studies. Longitudinal studies are needed to better understand the causal pathways linking social determinants to multimorbidity. Expanding the geographic scope of research and exploring how factors like gender, socioeconomic status, and location intersect will provide a more comprehensive understanding of multimorbidity in SSA. Including qualitative approaches can also help capture the lived experiences of older adults managing multiple chronic conditions. By addressing these gaps, future research can support the development of targeted policies and interventions, ultimately reducing the burden of multimorbidity and improving the health and well-being of older adults across the region.

## Data Availability

The data that support the findings of this systematic review were derived from the included studies, which are publicly available through their respective publishers and databases. The references for these studies were listed in the manuscript.
